# Higher Prevalence of Extended-Spectrum Cephalosporin-Resistant *Enterobacterales* in Dogs Attended for Enteric Viruses in Brazil Before and After Treatment with Cephalosporins

**DOI:** 10.3390/antibiotics10020122

**Published:** 2021-01-28

**Authors:** Marília Salgado-Caxito, Andrea I. Moreno-Switt, Antonio Carlos Paes, Carlos Shiva, Jose M. Munita, Lina Rivas, Julio A. Benavides

**Affiliations:** 1 Department of Animal Production and Preventive Veterinary Medicine, School of Veterinary Medicine and Animal Science, Sao Paulo State University, Botucatu 18618000, Brazil; ac.paes@unesp.br; 2 Millennium Initiative for Collaborative Research on Bacterial Resistance (MICROB-R), Santiago 7550000, Chile; andrea.moreno@uc.cl (A.I.M.-S.); munita.jm@gmail.com (J.M.M.); linarivas@udd.cl (L.R.); 3 Escuela de Medicina Veterinaria, Pontificia Universidad Católica de Chile, Santiago 8940000, Chile; 4 Faculty of Veterinary Medicine and Zootechnics, Universidad Cayetano Heredia of Peru, Lima 15102, Peru; carlos.shiva@upch.pe; 5 Genomics and Resistant Microbes Group, Facultad de Medicina Clinica Alemana, Universidad del Desarrollo, Santiago 7550000, Chile; 6 Departamento de Ecología y Biodiversidad, Facultad de Ciencias de la Vida, Universidad Andrés Bello, Santiago 8320000, Chile; 7 Centro de Investigación para la Sustentabilidad, Facultad de Ciencias de la Vida, Universidad Andrés Bello, Santiago 8320000, Chile

**Keywords:** antimicrobial resistance, antimicrobial prophylaxis, canine distemper, canine parvovirus, companion animals

## Abstract

The extensive use of antibiotics is a leading cause for the emergence and spread of antimicrobial resistance (AMR) among dogs. However, the impact of using antibiotics to treat viral infections on AMR remains unknown. In this study, we compared the prevalence of extended-spectrum cephalosporin-resistant *Enterobacterales* (ESCR-E) between dogs with a suspected infection of canine parvovirus (CPV) and canine distemper (CDV) before and after treatment with third-generation cephalosporins. We found a higher prevalence of ESCR-E faecal carriage in dogs suspected of CPV (37%) and CDV (15%) compared to dogs with noninfectious pathologies (9%) even prior to the start of their treatment. A 7-day course of ceftriaxone or ceftiofur administrated to CPV and CDV-suspected dogs substantially increased their ESCR-E faecal carriage during treatment (85% for CPV and 57% for CDV), and 4 weeks after the treatment ended (89% for CPV and 60% for CDV) when dogs were back in their households. Most of the observed resistance was carried by ESCR-*E. coli* carrying *bla*_CTX-M_ genes. Our results suggest the need to optimize prophylactic antibiotic therapy in dogs treated for a suspected viral infection to prevent ESCR-E emergence and spread in the community.

## 1. Introduction

Antimicrobial resistance (AMR) in companion animals is one of the major challenges for the treatment of infections in veterinary practice [1]. Part of the burden of AMR is attributed to extended-spectrum cephalosporin-resistant *Enterobacterales* (ESCR-E), considered as “global priority pathogen” due to the limited options available for their effective treatment in both humans and animals [2,3]. ESCR-E are increasingly reported among dogs [4,5,6,7,8]. Although most studies identified ESCR-E in commensal *E. coli*, horizontal resistance gene transfer can spread resistance to other pathogenic microorganisms and result in severe bacterial infections with reduced treatment options [9]. However, the drivers for the acquisition and dissemination of ESCR-E in commensal bacteria in both clinical and community settings remain poorly understood in dogs.

The extensive use of antibiotics is the main driver for the emergence of ESCR-E faecal carriage in dogs [10,11,12]. Several antibiotics of critical importance to human health are commonly used to treat bacterial infections in dogs including extended-spectrum cephalosporins (third and fourth generation) and fluoroquinolones [13]. For example, these antibiotics are the most commonly prescribed by European veterinarians, although a few small animal veterinary centres have an antimicrobial stewardship policy [14]. The correlation between the frequency of antimicrobial use and AMR is well documented in livestock [15]. However, to our knowledge, no similar study has been conducted in dogs, limiting our understanding of how to optimize the use of antibiotics to reduce the emergence and spread of AMR in the veterinary practice.

Other than using antibiotics to treat bacterial infections, infections caused by other pathogens such as viruses can also require the use of antibiotics. Canine parvovirus (CPV) and canine *morbillivirus* (canine distemper, CDV) are often treated with third-generation cephalosporins due to the severe host immunosuppression and a risk of sepsis by bacterial translocation [16,17,18,19,20,21,22]. Despite been considered as an effective prophylactic treatment for these viruses, the use of third-generation cephalosporins in dogs could increase the selective pressure for ESCR-E [23]. CPV and CDV are one of the main causes of mortality in dogs, particularly puppies, with high prevalence estimated in several countries including Brazil [24,25,26,27,28,29]. However, despite the common circulation of these viruses, no study to our knowledge has evaluated the effects of prophylactic antibiotic therapy in CPV and CDV infections on the faecal carriage of antibiotic-resistant bacteria among dogs. 

The spread of ESCR-E potentially emerging during the treatment into the community (e.g., to other household members) will depend on the duration of ESCR-E faecal carriage after treatment. The spread of ESCR-E will also depend on the supporting genetic material coding the ESCR. Resistance to broad-spectrum cephalosporins is often associated to the presence of extended-spectrum β-lactamases (ESBL) enzymes that can hydrolyse β-lactams antibiotics (i.e., penicillins, cephalosporins, and cephamycins), which is the main mechanism for ESCR-*E. coli* in dogs and cats [4,6,30,31,32]. Most ESBL genes spread by insertion on mobile genetic elements such as plasmids [32]. ESBL genes have been detected in isolates from dogs 3 days after administration of first-generation cephalosporins prior to surgical procedures [33]. In addition, faecal carriage of ESBL-*E. coli* has been detected for up to 3 months in dog faeces after intravenous treatment with cephalexin and cefovecin [12]. Thus, limited available evidence shows the potential for the spread of ESBL-*E. coli* after treatment. However, a few studies have monitored treated dogs for longer periods. In this study, we first compared the prevalence of ESCR-E faecal carriage in dogs with clinical signs of CPV or CDV infections with uninfected dogs before antibiotic therapy at the referral veterinary teaching hospital of the Sao Paulo State University (FMVZ-UNESP) of Botucatu, Brazil. Then, we tested whether the use of third-generation cephalosporins to treat dogs suspected with CPV and CDV infections increased the faecal carriage of ESCR-E in dogs returning to their household up to 14 weeks after treatment. 

## 2. Results

### 2.1. Dogs’ Characteristics

A total of 222 dogs were sampled from Botucatu (Southeast Brazil) (63%) and cities within a radius of approximately 350 Km (37%). CPV-suspected dogs were 5 months old on average (range: 1–30 months, median: 3), half (52%, 27/52) were male, and 56% were mixed breed. CDV-suspected dogs were 34 months old on average (range: 1–108 months, median: 24), half (50%, 20/40) were male, and 73% were mixed breed. Noninfected dogs were 58 months old on average (range: 1–120 months, median: 60); the majority were female (56.2%, 73/130) and 55% were purebred. The type of food provided to dogs by owners were majority kibble (noninfected dogs: 95%, 118/124; CPV-suspected dogs: 100%, 38/38; and CDV-suspected dogs: 94%, 30/32), but owners also provided raw (noninfected dogs: 8%, CPV-suspected dogs: 18%, CDV-suspected dogs: 16%,) and cooked meat/poultry (noninfected dogs: 63%; CPV-suspected dogs: 45%, CDV-suspected dogs: 66%). 

### 2.2. Prevalence of Faecal Carriage and Characterization of ESCR-E Isolated from Dogs before Antibiotic Therapy

The prevalence of ESCR-E (i.e., *E. coli* and *K. pneumoniae*) faecal carriage in dogs prior to their admission at the referral veterinary teaching hospital FMVZ-UNESP was 16.7% (37/222) (95% CI: 12–22%). The prevalence of ESCR-E faecal carriage in CPV-suspected dogs (36.5% (95% CI: 25–50%) (19/52)) was higher than in CDV-suspected dogs (15% (95% CI: 7–29%) (6/40)) (Pearson’s test, *p* < 0.05) and that in noninfected dogs (9.2% (95% CI: 5–16%) (12/130)) (Pearson’s test, *p* < 0.001) (Table 1). No statistically significant difference was found between CDV-suspected dogs and noninfected dogs (Pearson’s test, *p* = 0.46).

We recovered 49 ESCR-*E. coli* and 3 ESCR-*K. pneumoniae* isolates from 37 dogs sampled before antibiotic therapy (details on each isolate are given in Table S1). To avoid duplicating same strains, isolates from the same sample showing the same antimicrobial resistance pattern (antimicrobial resistance phenotype) and ESBL genes were excluded from further analysis. All isolates were resistant to ceftriaxone, cefpodoxime, and cefotaxime, 63.5% to aztreonam, 26.9% to ceftazidime, and 15.4% to cefoxitin (Table 1). No isolate was resistant to carbapenems. We found 5 antimicrobial resistance phenotypes among dogs suspected of CPV and CDV infection, and 3 of them were also observed in noninfected dogs. β-lactamases genes were detected in 67% (33/49) of ESCR-*E. coli* and in 100% of *K. pneumoniae* (3/3) isolates from 37 dogs (Table 2). *bla*_CTX-M_ was detected in 60% (31/52) of isolates, *bla*_TEM_ in 21% (11/52), and *bla*_SHV_ in 6% (3/52). The ESBL *bla*_CTX-M_ was predominately identified in isolates resistant to ceftriaxone, cefpodoxime, cefotaxime, and aztreonam.

### 2.3. Effect of Prophylactic Antibiotic Treatment

From the 92 dogs clinically diagnosed with CPV and CDV infections sampled and then treated with third generation of cephalosporins, we sampled 20 dogs during their 7-day treatment and 36 dogs 1–4 weeks after (Table 3). Half (52%) of the dogs followed died during the study period, 19 dogs could not be accessed either during or after treatment, and 5 dogs have subsequent negative results that discontinued their follow-up. Details of the follow-up are given in Table S2.

**Figure 1:**
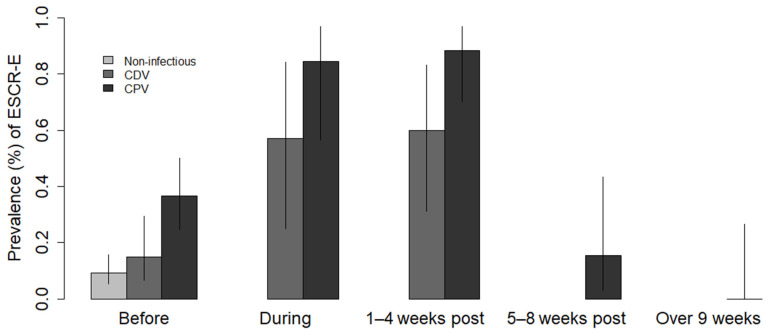
Prevalence of extended-spectrum cephalosporin-resistant *Enterobacterales* (ESCR-E) faecal carriage in dogs clinically diagnosed with canine parvovirus (CPV) and canine distemper (CDV) infections before, during, and after antibiotic therapy. The prevalence of dogs suspected of CPV and CDV infections was estimated at four periods after the start of antibiotic therapy with third-generation cephalosporin: “During” the 7-day course of ceftriaxone or ceftiofur, “1–4 weeks” post-treatment, “5–8 weeks” post-treatment, and “over 9 weeks” post-treatment. Before the treatment, the prevalence was compared to dogs without infectious diseases prior to their admission to the hospital for other health issues.

The prevalence of ESCR-E faecal carriage in dogs significantly increased during the 7-day course of treatment from 36.5% to 84.6% (95% CI: 56–97%) (11/13) in CPV-suspected dogs (Pearson’s test, *p* < 0.01) and from 15% to 57.1% (95% CI: 25–84%) (4/7) in CDV-suspected dogs (Pearson’s test, *p* < 0.05) (Figure 1). This prevalence remained high 1–4 weeks after the treatment in both CPV-suspected (88.5% (95% CI: 70–97%) (23/26)) and CDV-suspected dogs (60% (95% CI: 31–83%) (6/10)). Due to the high mortality of CDV-suspected dogs, only CPV-suspected dogs were monitored more than 9 weeks after treatment. The prevalence of ESCR-E faecal carriage in CPV-suspected dogs significantly decreased 5–8 weeks after treatment (15.4% (95% CI: 3–43.5%) (2/13)) (Pearson’s test, *p*<0.001) compared to 1–4 weeks. No ESCR-*E. coli* was detected in the 10 dogs monitored more than 9 weeks after treatment. 

We obtained 17 ESCR-*E. coli* and 3 ESCR-*K. pneumoniae* isolates from the 15 infected dogs sampled during antibiotic therapy, 60 ESCR-*E. coli* and 8 ESCR-*K. pneumoniae* isolates from the 29 infected dogs sampled 1–4 weeks after treatment, and 2 ESCR-*E. coli* from 2 infected dogs sampled 5–8 weeks after treatment (Table 1). All isolates obtained during and after the treatment were resistant to ceftriaxone, cefpodoxime, and cefotaxime, 61.1% to aztreonam, 30% to ceftazidime, and 16.7% to cefoxitin. No isolate was resistant to carbapenems. The five antimicrobial resistance phenotypes of isolates from dogs sampled before treatment were also found during and after treatment. Only one new antimicrobial resistance phenotype (resistance to ceftriaxone, cefpodoxime, cefotaxime, and cefoxitin) observed 1–4 weeks after treatment in 2 isolates of CPV-suspected dogs was not previously detected. 

## 3. Discussion

Despite the common use of extended-spectrum cephalosporins to treat enteric viruses, their impact on the prevalence of ESCR-E faecal carriage in dogs has not been previously studied. We found that the faecal carriage of ESCR-E was higher in CPV-suspected dogs compared to CDV-suspected dogs or noninfected dogs prior to their admission at the veterinary university hospital in Botucatu, Brazil. During the 7-day course of third-generation cephalosporin, the prevalence of ESCR-E faecal carriage increased by more than 50% in both CPV-suspected and CDV-suspected dogs, remained high up to 4 weeks after treatment, and could still be detected in dogs for up to 7 weeks post-treatment. A diversity of antibiotic phenotypes was observed, and the majority of the observed in ESCR-E was associated with the presence of *bla*_CTX-M_ genes. 

Secondary bacterial infections frequently worsen the prognosis of enteric viruses such as CPV and CDV, requiring prophylactic antibiotic therapies including third-generation cephalosporins [16,21,22,34,35,36,37]. Our study showed that the use of ceftriaxone or ceftiofur increased faecal prevalence of ESCR-E in hospitalized dogs during the treatment and remained 4 weeks after hospital discharge. Ceftiofur and ceftriaxone are similar third-generation cephalosporins although ceftiofur has been developed exclusively for animal treatment [38]. Both cephalosporins contain an *oxyimino-aminothiazole* group (also found in other antimicrobials such as cefpodoxime, ceftazidime, cefotaxime, ceftizoxime, and aztreonam), which is hydrolysed by extended-spectrum β-lactamases conferring resistance to these β-lactams antibiotics [38,39]. Several studies have shown an increase in AMR *E. coli* in animals after selection pressure by use of β-lactams and fluoroquinolones [11,12,15,40,41,42,43]. For example, parenteral antibiotic therapy with extended-spectrum cephalosporins and hospitalization longer than 6 days increased the faecal carriage multidrug-resistant *E. coli* during hospitalization [44]. The presence of ESCR-E faecal carriage in the hospitalized dogs in our study suggests either independent circulation of these strains in the community or a selection in the hospital during treatment that is then spread in dogs of the community. Although we have not evaluated the clinical impact of the emergence of ESCR-E during treatment for enteric viruses, nosocomial infections by resistant bacteria in veterinary hospitals are been increasingly reported in small animal practices worldwide and several approaches should be used to reduce the risks [45,46,47]. Ensuring an appropriate use of third-generation cephalosporins may include de-escalation of antibiotic therapy or shorter duration of treatment when the clinical improvement of patients is observed [48]. However, the efficiency of these approaches should be first tested in veterinary practices. Optimizing antibiotic use without compromising its efficacy is particularly important when treating CPV-infected dogs because bacteremia and sepsis are commonly observed due to the loss of the intestinal barrier and translocation of Gram-negative bacteria [16,21,22].

Our results suggest that ESCR-*E. coli* faecal carriage in CPV-suspected dogs can last up to 7 weeks after treatment. Similar studies have shown that treatment with antibiotics increases the prevalence and persistence of AMR *Enterobacterales* [10,11]. For example, treatment with amoxicillin and clavulanic acid has been associated with ESCR-*E. coli* faecal carriage 1 month after treatment [12]. Alternatively to persistence, ESCR-*E. coli* may be reacquired after treatment from external sources, which could be evaluated in future molecular studies. Overall, our study calls for increasing awareness regarding the potential spread of ESCR-E in clinical and community settings caused by treatments of enteric viruses with antibiotics.

A total of six antimicrobial resistance profiles were obtained in our study. One profile was only detected after treatment. This new profile could reflect the emergence of new antibiotic resistance associated with the selection impose by the treatment or a low detection probability of all the resistant profiles before treatment given our limited sample size. Resistance to ceftriaxone, cefpodoxime, cefotaxime, and aztreonam was the main profile observed, which is often associated with ESBL production [32]. In fact, the majority (60%) of ESCR-E isolates (i.e., *E. coli* and *K. pneumoniae*) from CPV-suspected dogs and noninfected dogs (67%) carried *bla*_CTX-M_ genes, followed by CDV-suspected dogs (43%). CTX-M genotype was the most prevalent genotype found among ESCR-E isolates, confirming the spread of ESBL in dogs [11,49,50,51,52]. Differences in the antimicrobial resistance phenotype between CTX-M-positive isolates might reflect the variety of CTX-M groups (i.e., CTX-M-1, CTX-M-2, CTX-M-8, CTX-M-9, and CTX-M-25) that may present different hydrolysing activities for cephalosporins [32,53]. In addition, the concomitant presence of AmpC genes (chromosomally encoded or plasmid-mediated) results in cefoxitin resistance and can mask the presence of ESBL [54,55]. Although our PCR protocol included primers specifically designed to detect common extended-spectrum β-lactamases genes, future molecular analyses including sequencing of these resistance genes (e.g., *bla*_SHV_ and *bla*_TEM_) will confirm if they are ESBL genes and identify their variant. No β-lactamase genes were detected in 16 ESCR-E isolates but the phenotypic resistance observed may be due to other β-lactamases not tested in the current study (e.g., CMY, PER, and OXA), mutations in the chromosomal AmpC gene, efflux pumps, or pore deficiencies [56]. Therefore, further molecular studies such as whole-genome sequencing could help identifying all that antibiotic resistance mechanisms present among these bacteria. 

To our knowledge, this is the first study showing a higher prevalence of faecal carriage of antibiotic-resistant bacteria in dogs presenting clinical signs of enteric viruses. Brazil has a high prevalence of CPV and CDV, and these viruses are the leading cause of mortality related to infectious diseases in dogs with an incidence above 45% in some areas with low vaccine coverage [57,58]. Thus, increase in ESCR-*E. coli* after treatment could have important implications for the spread of ESCR-*E. coli* among dogs in Brazil. CPV and CDV alter the host microbiota [16,17,34,59,60] and could be influencing the gut colonization by antibiotic-resistant bacteria such as ESCR-E. Since the immune system regulates the gut microbiota [61], host immunosuppression provoked by these viruses may favour mechanisms expressing different genes including ESBL genes. For instance, immunosuppressive treatments with a combination of prednisolone, mycophenolate mofetil, and tacrolimus increased the population of uropathogenic *E. coli* in treated humans [62]. CPV has a strong affinity of rapidly dividing cells causing destruction of crypt intestinal epithelial cells, which might generate a higher impact on the microbiota, including antimicrobial-resistant bacteria. Alternatively, other explanations for the observed outcome could include exposure to antibiotics by infected dogs prior to the period established in our inclusion criteria (3 months). However, this hypothesis is not supported by the observation that most infected dogs were puppies and thus, were probably not exposed directly to antibiotics’ treatment. Thus, the mechanisms behind the higher prevalence of ESCR-E in CPV-suspected dogs remain unclear. Other explanations could include dogs been intensely exposed to antibiotic-resistant bacteria from other sources such as humans or livestock, and the puppies’ mothers been a source of antibiotic-resistant bacteria. For example, adult dogs leaving in regions with low vaccine coverage favouring the acquisition of CPV [57] could be more likely to have concomitant diseases and/or a history of receiving antimicrobials, which could indirectly expose their puppies. 

Several limitations of our study could encourage future research on the role of enteric viruses along with antimicrobial prophylaxis in the emergence of AMR. For examples, although most differences in prevalence between dog populations were statistically significant, the number of followed dogs after treatment was substantially reduced by the high mortality of infected dogs (52%) and difficulty to access dogs at their household. Therefore, the lack of detection of dogs with ESCR-E 50 days after treatment could be related to a low detection power due to our low sample size (*n* = 5) and could be further studied in future research. Furthermore, since the excretion of antibiotic-resistant bacteria hosted on the dog’s intestinal microbiota can be shed intermittently on faeces, a lack of detection within a faecal sample does not necessarily indicate the absence of faecal carriage in the sampled dog. In addition, we were unable to follow uninfected dogs to confirm that the observed increased in ESCR-*E. coli* prevalence was associated with prophylactic treatment with antibiotics and no other factors such as colonization at the hospital after visit or unknown interactions between CPV or CDV and antibiotic-resistant bacteria. Further studies covering these limitations could explore in more detail both the effect of antibiotic prophylactic treatment and enteric viruses in relation to antimicrobial resistance. The potential misdiagnoses of a bacterial infection cannot be ruled out and require further studies using molecular detection of CPV and CDV to confirm the clinical diagnostics, which was not available at our hospital. However, pathognomonic clinical signs of CDV and CPV such as myoclonus (CDV) or acute haemorrhagic diarrhoea along with intense leukopenia on the peripheral blood (CPV) observed in these animals suggest that misdiagnosis should only represent a small percentage of our population. Furthermore, we cannot exclude the chances of coinfections with other enteropathogens; however, these clinical signs also suggest that illness severity was associated to the presence of these enteric viruses and not aggravated by another agent. Finally, molecular typing of resistant bacteria and detection of mobile genetic elements (e.g., plasmids) will help to understand whether the increase in ESCR-E after treatment is due to maintenance of the same ESCR-E strain, infection with new bacteria or transfer of genetic material across strains conferring antibiotic resistance. 

## 4. Materials and Methods 

### 4.1. Comparison of Prevalence of Dogs Carrying ESCR-E before Antibiotic Therapy

Between August and December 2018 at the referral veterinary teaching hospital of the Sao Paulo State University (FMVZ-UNESP) of Botucatu (Southeast Brazil), rectal swabs were collected from dogs suspected of CPV (*n* = 52) and CDV (*n* = 40). In addition, we also sampled 130 dogs classified as noninfected dogs by the veterinarian collecting the sample as a control group. Dogs were physically examined by a veterinarian and the owners were asked about the previous use of antibiotic in their dogs within the last 3 months. Two exclusion criteria were used: i) the use of antimicrobials within 3 months before sampling and ii) dogs more than 10 years old.

Dogs attended in the Animal Infectious Diseases sector presenting haemorrhagic diarrhoea, vomiting, dehydration, and intense leukopenia on peripheral blood [16] were diagnosed with CPV infection by the veterinarian attending while signals of nonhaemorrhagic diarrhoea, respiratory disorders, nasal and ocular discharges, hyperkeratosis, and neurological manifestation (i.e., myoclonus) were diagnosed as CDV infection [34]. To be included in this study, infected dogs had to present all the described clinical signs to guarantee homogeneity among CPV and CDV groups and to attest the severity of the illness. Noninfected dogs were attended in the sectors of Cardiology, Nephrology, Neurology, Surgery, or Ophthalmology and showed absence of clinical signs of infectious diseases and/or gastrointestinal disorders (dogs suspected of infection even without symptoms such as gastrointestinal alterations or respiratory disorders were not included).

The sample size required to estimate the prevalence of noninfected dogs was determined using Epi Info 7.2.2.6 TM [63]. Based on an expected prevalence of ESCR-*E. coli* of 9% estimated in a previous study conducted in Brazil [49], a dog population estimated in Botucatu of 27,735 dogs [64], an acceptable margin of error of 5% and confidence interval of 95%, the estimated sample size was 125 animals. A sampling of dogs suspected of viral infections was based on convenience, enrolling all suspected dogs with CPV and CDV admitted to the hospital in 5 months, considering that many dies before or during the treatment. 

### 4.2. Faecal Prevalence of ESCR-E During and After Antibiotic Therapy With Third-Generation Cephalosporins in Dogs Suspected of CPV and CDV Infections

CPV and CDV suspected dogs were treated with parenteral ceftriaxone (30 mg/Kg) or ceftiofur (7.5 mg/Kg) every 24 h for 7 days. To test how the prevalence of ESCR-E in dogs changes after treatment, all treated dogs were sampled in the following periods: (1) before administration of third-generation cephalosporin, (2) during the 7-day course of treatment, and (3) after antibiotic therapy between the first and fourth week (1–4 weeks post-treatment). In dogs where ESCR-E was detected during or after treatment, subsequent sampling was done (4) between the fifth and eighth week (5–8 weeks post-treatment) and (5) above the ninth week (over 9 weeks post-treatment). The number of samples after antibiotic therapy varied from one to three per dog and sampling of dogs was not paired (Table S2). This study was approved by the Ethical Committee in Animal Use (CEUA) of the FMVZ-UNESP/Botucatu under protocol: 0090/2018 (registration number on CONCEA–National Council for Animal Control and Experimentation: CIAEP/CONCEA no. 01.0115.2014–05/06/2014), and all owners signed a consent form for inclusion of their dogs.

### 4.3. Microbiology Analysis 

Rectal swabs were screened for ESCR-E using MacConkey agar supplemented with 2 μg/mL cefotaxime (Oxoid, Hampshire, England) and incubated at 37 °C for 48 h to select potential ESBL/AmpC-producing isolates [65]. *E. coli* strain containing the *bla*_CTX-M15_ gene (provided by the Microbiology Laboratory of Institute of Biosciences-UNESP) and a non-resistant *E. coli* strain (donated by the Microbiology Laboratory of the Veterinary Teaching Hospital of FMVZ-UNESP) were used as positive and negative controls. Up to three isolates morphologic compatible with *E. coli* or *K. pneumoniae* were randomly selected in the plate and then confirmed by matrix-assisted laser desorption ionization-time of flight mass spectrometry (MALDI-TOF MS, BioMérieux, Marcy l’Etoile, France) at the Genomics and Resistant Microbes (GeRM) Group of the Millennium Initiative for Collaborative Research On Bacterial Resistance (MICROB-R), in Santiago, Chile. Other species identified were excluded from the further analysis (i.e., *Escherichia fergusonii*, *Raoultella ornithinolytica*, *Raoultella planticola*, and *Citrobacter freundii*).

According to the CLSI M100:28ED, cefpodoxime (10 µg) with inhibition zone ≤ 17 mm, ceftazidime (30 µg) with inhibition zone ≤ 22 mm, aztreonam (30 µg) with inhibition zone ≤27 mm, cefotaxime (30 µg) with inhibition zone ≤ 27 mm, and ceftriaxone (30 µg) with inhibition zone ≤25 mm of *E. coli* isolates may indicate ESBL production. To select extended-spectrum cephalosporins-resistant isolates, particularly the ESBL producers, we tested ceftriaxone (30 µg), cefpodoxime (10 µg), cefotaxime (30 µg), ceftazidime (30 µg), aztreonam (30 µg), and cefoxitin (30 µg) by the disk diffusion method according to the CLSI [65]. We preferred to include a combination of these antibiotics instead on only one of them, to improve the detection of ESBL production. To assess co-resistance to carbapenems, we also tested susceptibility to imipenem (10 µg), meropenem (10 µg), and ertapenem (10 µg). Breakpoints and a quality control *E. coli* ATCC25922 strain was used during each assay as recommended by CLSI [65]. A multiplex PCR protocol was performed to detect β-lactamases genes (*bla*_CTX-M_, *bla*_SHV_, and *bla*_TEM_) in ESCR-*E. coli* and in ESCR-*K. pneumoniae* isolated before antibiotic therapy using primers previously published [66,67]. Primers sequence and PCR conditions are given in the additional data (Table S3). *E. coli* strain SCL-1290 of MICROB-R repository containing these three genes was used as a positive control. 

### 4.4. Statistical Analysis

The prevalence of dogs colonized by ESCR-E, referred here as the number of individuals with at least one positive isolate of ESCR-*E. coli* or ESCR-*K. pneumoniae* over the total number of sampled animals, was reported with a 95% confidence interval using the *binom.confint* function (Agresti–Coull method) in the *binom* package in R 3.6.1 [68]. Differences in prevalence were tested using the Pearson test’s chi-squared in R.

## 5. Conclusions

Our results show that prophylactic antibiotic therapy in dogs clinically diagnosed with enteric viruses (i.e., CPV and CDV) can play an important role in the dissemination of ESCR-E in clinical settings and the community. Although antimicrobial prophylaxis in these diseases is necessary, we highlight the importance of optimizing prophylactic antibiotic therapy in infected dogs by prioritizing first or second generation of cephalosporin in mild cases and third-generation cephalosporins only for life-threatening one. Even if new classes of antimicrobial agents are developed, they are unlikely to be available for veterinary medicine in the short term. Therefore, emergence and persistence of resistance to broad-spectrum cephalosporins observed in this study after treatment with third-generation of cephalosporins stress the need for widespread to veterinarians targeting the necessity to maintain the effectiveness of current antibiotic therapies. In addition, our findings suggest that the high prevalence of CPV and CDV may be aggravating the spread of ESCR-*E. coli* among dogs in Brazil, where vaccination against canine viruses with exception of rabies is not mandatory [57]. Therefore, reducing the circulation of CPV and CDV by improving vaccination coverage could help to reduce the dissemination of ESCR-E. Our results also call for further studies to identify the mechanisms behind the observed association between enteric viruses and faecal carriage of antimicrobial-resistant bacteria.

## Figures and Tables

**Table 1:** Clinical signs, prevalence of extended-spectrum cephalosporin-resistant *Enterobacterales* (ESCR-*E*) and antimicrobial resistance profiles isolated from faeces of dogs clinically diagnosed with canine parvovirus (CPV) and canine distemper (CDV) infections before and after prophylactic antibiotic therapy with third-generation cephalosporin and from dogs with noninfectious diseases.

Variable	CPV-Suspected Dogs	CDV-Suspected Dogs	Noninfected Dogs
Main clinical signs	Haemorrhagic diarrhoea Vomiting Intense dehydration	Nonhaemorrhagic diarrhoea Respiratory disorders Neurological signs	Signs of noninfectious diseases
Prevalence BEFORE treatment	36.5% (19/52) ^1^ (95% CI: 25–50%)	15% (6/40) (95% CI: 7–29%)	9.2% (12/130) (95% CI: 5–16%)
AMR profiles BEFORE treatment	*E. coli* CroCpdCtx (*n* = 5) ^2^ CroCpdCtxAtm (*n* = 12) CroCpdCtxCazAtm (*n* = 1) CroCpdCtxCazFox (*n* = 2) CroCpdCtxCazFoxAtm (*n* = 4) *K. pneumoniae* CroCpdCtxAtm (*n* = 1) CroCpdCtxCazAtm (*n* = 2)	*E. coli* CroCpdCtx (*n* = 3) CroCpdCtxAtm (*n* = 1) CroCpdCtxCazAtm (*n* = 1) CroCpdCtxCazFox (*n* = 1) CroCpdCtxCazFoxAtm (*n* = 1)	*E. coli* CroCpdCtx (*n* = 8) CroCpdCtxAtm (*n* = 8) CroCpdCtxCazAtm (*n* = 2)
Prevalence DURING treatment	84.6% (11/13) (95% CI: 56–97%)	57.1% (4/7) (95% CI: 25–84%)	N/A
AMR profiles DURING treatment	*E. coli* CroCpdCtx (*n* = 2) CroCpdCtxAtm (*n* = 7) CroCpdCtxCazAtm (*n* = 2) *K. pneumoniae* CroCpdCtx (*n* = 1) CroCpdCtxCazAtm (*n* = 2)	*E. coli* CroCpdCtx (*n* = 1) CroCpdCtxAtm (*n* = 2) CroCpdCtxCazAtm (*n* = 1) CroCpdCtxCazFox (*n* = 1) CroCpdCtxCazFoxAtm (*n* = 1)	N/A
Prevalence 1–4 WEEKS after treatment	88.5% (23/26) (95% CI: 70–97%)	60% (6/10) (95% CI: 31–83%)	N/A
AMR profiles 1–4 WEEKS after treatment	*E. coli* CroCpdCtx (*n* = 17) CroCpdCtxAtm (*n* = 18) CroCpdCtxCazAtm (*n* = 5) CroCpdCtxCazFox (*n* = 5) CroCpdCtxCazFoxAtm (*n* = 4) CroCpdCtxFox (*n* = 2) * K. pneumoniae CroCpdCtx (*n* = 3) CroCpdCtxAtm (*n* = 2) CroCpdCtxCazAtm (*n* = 2) CroCpdCtxCazFoxAtm (*n* = 1)	*E. coli* CroCpdCtx (*n* = 2) CroCpdCtxAtm (*n* = 4) CroCpdCtxCazAtm (*n* = 2) CroCpdCtxCazFoxAtm (*n* = 1)	N/A
Prevalence 5-8 WEEKS after treatment	15.4% (2/13) (95% CI: 3–43.5%)	N/A	N/A
AMR profiles 5-8 WEEKS after treatment	*E. coli* CroCpdCtx (*n* = 1) CroCpdCtxAtm (*n* = 1)	N/A	
Prevalence over 9 WEEKS after treatment	0% (0/10)	N/A	N/A

^1^ Number of positive dogs over the total number of sampled dogs. ^2^ Number of isolates. * Antimicrobial resistance phenotype only observed after treatment. Abbreviations: Cro—ceftriaxone, Cpd—cefpodoxime, Ctx—cefotaxime, Caz—ceftazidime, Fox—cefoxitin, Atm—aztreonam, N/A—data not available.

**Table 2:** Resistance profile and β-lactamases genes identified from ESCR-*E. coli* and ESCR-*K. pneumoniae* isolates of 19 CPV-suspected dogs, 6 CDV-suspected dogs, and 12 uninfected dogs before antibiotic therapy.

Dog ID	Bacteria Species	Strain	AMR Profile	CTX-M ^1^	SHV	TEM
CPV-2	*E. coli*	MS1_001	CroCpdCtxCazFox	-	-	+
CPV-3	*E. coli*	MS1_018	CroCpdCtxCazFox	-	-	+
CPV-4	*E. coli*	MS1_022	CroCpdCtxCazFoxAtm	-	-	+
CPV-5	*E. coli*	MS1_034	CroCpdCtxCazFoxAtm	-	-	-
CPV-5	*E. coli*	MS1_035	CroCpdCtx	-	-	-
CPV-5	*K. pneumoniae*	MS1_036	CroCpdCtxCazAtm	+	+	+
CPV-5	*K. pneumoniae*	MS1_038	CroCpdCtxAtm	+	+	+
CPV-6	*E. coli*	MS1_052	CroCpdCtx	-	-	+
CPV-6	*E. coli*	MS1_053	CroCpdCtxCazFoxAtm	-	-	-
CPV-6	*E. coli*	MS1_054	CroCpdCtxAtm	-	-	-
CPV-7	*E. coli*	MS1_067	CroCpdCtx	-	-	+
CPV-7	*E. coli*	MS1_069	CroCpdCtxAtm	-	-	-
CPV-8	*E. coli*	MS1_083	CroCpdCtxCazFoxAtm	-	-	-
CPV-13	*E. coli*	MS1_111	CroCpdCtxAtm	+	-	-
CPV-14	*E. coli*	MS1_120	CroCpdCtxAtm	+	-	-
CPV-15	*E. coli*	MS1_123	CroCpdCtxAtm	+	-	-
CPV-16	*E. coli*	MS1_129	CroCpdCtxAtm	+	-	-
CPV-26	*E. coli*	MS1_152	CroCpdCtxAtm	+	-	-
CPV-41	*E. coli*	MS1_186	CroCpdCtx	+	-	-
CPV-42	*E. coli*	MS1_192	CroCpdCtxAtm	+	-	-
CPV-42	*E. coli*	MS1_194	CroCpdCtx	+	-	-
CPV-43	*E. coli*	MS1_198	CroCpdCtxCazAtm	+	-	-
CPV-43	*E. coli*	MS1_199	CroCpdCtxAtm	+	-	-
CPV-44	*E. coli*	MS1_204	CroCpdCtxAtm	+	-	-
CPV-45	*K. pneumoniae*	MS1_210	CroCpdCtxCazAtm	+	+	+
CPV-46	*E. coli*	MS1_213	CroCpdCtxAtm	+	-	-
CPV-50	*E. coli*	MS1_223	CroCpdCtxAtm	+	-	-
CDV-7	*E. coli*	MS1_234	CroCpdCtx	+	-	-
CDV-21	*E. coli*	MS1_249	CroCpdCtx	-	-	-
CDV-27	*E. coli*	MS1_252	CroCpdCtx	-	-	-
CDV-28	*E. coli*	MS1_258	CroCpdCtxAtm	+	-	-
CDV-29	*E. coli*	MS1_261	CroCpdCtxCazFox	-	-	-
CDV-29	*E. coli*	MS1_262	CroCpdCtxCazFoxAtm	-	-	-
CDV-37	*E. coli*	MS1_267	CroCpdCtxCazAtm	+	-	-
NI-11	*E. coli*	MS1_270	CroCpdCtxAtm	-	-	-
NI-11	*E. coli*	MS1_271	CroCpdCtx	-	-	-
NI-19	*E. coli*	MS1_273	CroCpdCtx	-	-	-
NI-29	*E. coli*	MS1_276	CroCpdCtxAtm	+	-	+
NI-29	*E. coli*	MS1_278	CroCpdCtxCazAtm	+	-	+
NI-43	*E. coli*	MS1_279	CroCpdCtx	-	-	-
NI-44	*E. coli*	MS1_282	CroCpdCtx	-	-	-
NI-44	*E. coli*	MS1_283	CroCpdCtxAtm	+	-	-
NI-59	*E. coli*	MS1_285	CroCpdCtxAtm	+	-	-
NI-72	*E. coli*	MS1_287	CroCpdCtx	+	-	-
NI-78	*E. coli*	MS1_290	CroCpdCtx	+	-	-
NI-78	*E. coli*	MS1_291	CroCpdCtxAtm	+	-	-
NI-93	*E. coli*	MS1_293	CroCpdCtxAtm	+	-	-
NI-94	*E. coli*	MS1_296	CroCpdCtxAtm	+	-	-
NI-94	*E. coli*	MS1_298	CroCpdCtx	+	-	+
NI-97	*E. coli*	MS1_299	CroCpdCtx	-	-	-
NI-106	*E. coli*	MS1_302	CroCpdCtxAtm	+	-	-
NI-106	*E. coli*	MS1_303	CroCpdCtxCazAtm	+	-	-

^1^ Extended-spectrum β-lactamase, (+): detection of the gene, (-): no detection of the gene. Abbreviations: Cro—ceftriaxone, Cpd—cefpodoxime, Ctx—cefotaxime, Caz—ceftazidime, Fox—cefoxitin, Atm—aztreonam.

**Table 3:** Design and sample size of our longitudinal study tracking ESCR-E before and after antibiotic treatment.

Number of Dogs/Sampling Period	Before	During	1–4 Weeks after Treatment	5–8 Weeks after Treatment	Over 9 Weeks after Treatment
Number of CDV-suspected dogs	40	7	10	–	–
Number of CPV-suspected dogs	52	13	26	13	10
Median of sampling day resulting in ESCR-E isolates	–	4	14	50	–
Number of deaths	–	22	31	48	48
Number of dogs that were not accessed for sampling	–	49	21	22	3
Number of dogs sold	–	1	4	4	6
Number of dogs with subsequent negative results	–	–	–	5	5

## Supplementary Materials

The following are available online at https://www.mdpi.com/2079-6382/10/2/122/s1, Table S1: Characterization of isolates recovered from dogs clinically diagnosed with canine parvovirus (CPV) and canine distemper (CDV) infections and from dogs with noninfectious diseases before antibiotic therapy and of isolates recovered from dogs suspected of CPV and CDV infections after of antibiotic therapy with third-generation cephalosporin started; Table S2: Sampling details of the follow-up of the dogs suspected of CPV and CDV infections after of antibiotic therapy with third-generation cephalosporin; Table S3. Sequences, melting temperature, and amplicon size of primers used for detection of CTX-M-type, SHV-type, and TEM-type genes.
